# Comparing DTL microfiber and Neuroline skin electrode in the Mini Ganzfeld ERG

**DOI:** 10.1186/s12886-016-0311-4

**Published:** 2016-08-05

**Authors:** Anastasia Lapkovska, Anja M. Palmowski-Wolfe, Margarita G. Todorova

**Affiliations:** Department of Ophthalmology, University of Basel, Mittlere Strasse 91, CH-4031 Basel, Switzerland

**Keywords:** DTL microfiber electrode, Neuroline surface electrode, Full-field ERG, Mini Ganzfeld ERG, ISCEV protocol

## Abstract

**Background:**

In infant ERG recordings skin electrodes frequently result in a better compliance. In order to assess the quality of such recordings, we compared the recording characteristics of DTL microfiber and Neuroline surface electrodes using a modified ISCEV protocol in the Mini Ganzfeld ERG.

**Methods:**

A prospective cohort study on healthy adult subjects was conducted at the Department of Ophthalmology, University of Basel, Switzerland. Thirty healthy volunteers were tested. The microfiber electrode (DTL Plus Electrode) was placed across the cornea, above the lower eyelid. The Neuroline skin electrode was placed on the surface of the lower lid on the opposite eye. The eye on which each electrode type was placed was randomised.

Amplitudes of the rod, standard combined, standard flash cone, light-adapted 3.0 Hz flicker and red cone responses were analysed, as well as their respective implicit times.

**Results:**

Both electrode recordings showed the same waveform characteristics. Responses with the Neuroline electrode were significantly weaker than those from the DTL electrode. Amplitudes of the rod, standard combined, standard flash cone, light-adapted 3.0 Hz flicker and red cone responses were up to four times larger when recorded with the DTL electrode (*p* < 0.005, ANOVA). Implicit times of the red cone ERGs were slightly faster for the Neuroline skin electrode recordings (*p* ≤ 0.039).

**Conclusions:**

Comparison of full-field ERG recordings with microfiber DTL and Neuroline skin electrodes showed that DTL electrodes produce larger ERGs. Hence, we provide evidence that both electrode types allow successful full-field ERG recording, although separate normative data for both electrodes are necessary.

## Background

Full-field ERG is a widely used diagnostic tool for evaluation of the functional integrity of the retina. According to the first standardized protocol for ERG recordings of the International Standardization Committee and the following revised ISCEV standards, electrodes for recording the standard full-field ERG should contact the cornea or the bulbar conjunctiva. Recommended are contact lens electrodes, conductive fibres and foils, conjunctival loop electrodes, and corneal wicks [1–3]. Contact lens electrodes are preferable as they are more stable, thus, providing highest amplitudes and a higher signal-to-noise ratio [2]. However, in small children or disabled patients recording with a contact lens electrode is not always possible [4]. Moreover, in clinical settings, the fiber DTL electrode (Dawson, Trick, and Litzkow) is tolerated better than a contact lens Henkes electrode [5].

As exemplified in a survey of paediatric visual electrophysiology among members of ISCEV, more than one electrode type (124 % of sample) has been used. Here, the majority has preferred the standardized by ISCEV electrodes (94 % of sample) as: contact lens (61 % of sample), thread or foil (30 % of sample), and HK loop (3 % of sample). Only about one third of participants (29 %) have used also skin electrodes, and about half of them (12 %) preferred only skin electrodes [6].

In order to obtain artefact-free ERG recordings the subject’s cooperation is necessary. Therefore, in anxious, uncooperative, younger children, or in disabled patients general anaesthesia might be indicated [4, 7]. Analysis of such recordings under anaesthesia is quite difficult as, despite some contradictory results [8], anaesthetic agents may influence the ERG [9, 10]. In addition, the risk of repeated use of anesthetics with cognitive development should be taken into account. Furthermore, there are ethical problems with recording normative data on healthy children under pharmacological sedation. The possibility of recording ERGs without cornea contact has further advantages. Despite better subjects’ comfort, the DTL electrode does not obscure optics of the eye and shows reduced electrode impedance.

Nowadays, skin electrodes provide an alternative to contact lens or DTL electrodes. However, the skin electrode is not recommended by the ISCEV standard [1, 2], as the proper and stable positioning of the skin electrode on the surface of the lowed lid, blinking artefacts, as well as the direction of gaze have an impact on the recording. [11, 12]. Hence, when appropriately positioned, the ERG responses with skin electrodes, even with slight session-to-session variability in amplitudes, provide reproducible and reliable measurements [13–16]. Given the fact that children are more tolerant to skin electrodes [11, 12], we aimed to investigate on whether non-cornea contact electrodes (Neuroline skin electrode and DTL electrode) can be used to record standard full-field ERG responses.

The Mini Ganzfeld ERG hand-held flash stimulator produces full-field stimulation. Recently, hand-held ERG devices gained much attention in animal studies [17, 18] and for ERG recording in children under general anaesthesia [19]. The hand-held ERG devices have also been used to monitor retinal function during and after selective ophthalmic artery chemotherapy infusion in retinoblastoma [17, 20, 21], but also to elicit S-cone retinal responses [22]. The closer positioning of the flash stimulator to the eye provides stable flash luminance during the recording session. It produces also a flash, bright enough, to pass through the pupil even when the eye is in extreme positions. Since it is hand-held, the infants can rest comfortably in their strollers or on mum’s lap. Changing head positions can be followed easily, especially in infants. Thus, the Mini Ganzfeld has proved to be as reliable, as tabletop ERG recordings [23].

The aim of the study is to compare the recording characteristics of the Neuroline skin electrode responses to those recorded using the universally recognized DTL electrode on healthy adult subjects using a hand-held Mini Ganzfeld stimulator following the test order of the standard ISCEV protocol (2008) [2] modified with additional red cone ERG flashes.

## Methods

A prospective cohort study on healthy adult subjects was conducted in order to assess the recording characteristics of DTL microfiber and Neuroline surface electrodes using the standard ISCEV protocol (2008 updated) in the Mini Ganzfeld ERG. For complicity of the data, our laboratory records on routine basis red cone ERG responses. Therefore, four sets of red cone flashes superimposed after standard 2008 ISCEV protocol were recorded and evaluated, as well.

### Subjects

A total of 30 healthy volunteers (24 female, 6 male), mean age: 39.97 (SD ± 11.63*;* age range: 19-60 years) were tested between January 2013 and December 2013. Ophthalmologic examination was completed prior to the ERG examination. All healthy subjects fulfilled the following inclusion criteria: best corrected Snellen visual acuity of better than 0.9, intraocular pressure (IOP) under 21 mmHg. Exclusion criteria were hyperopia or myopia greater than 6dpt, any ocular pathology, previous ocular surgery, as well as systemic diseases and medication such as diabetes, hypertension, Parkinson disease, centrally acting medication, which might influence the full-field ERG recordings. Subjects with amblyopia, and nystagmus were excluded, as well.

Procedures followed the tenets of the Declaration of Helsinki. The study was approved by the local Ethics Committee of Basel (“Ethical committee of both Basel”, EKBB Nr: 33/12). The insurance was covered by HDI Gerling, Basel (Police Nr: 01055241-14003, study Nr: 12.023). A written informed consent was signed from all participants before the commencement of the examination.

### Electrodes and recordings

**Fig. 1:**
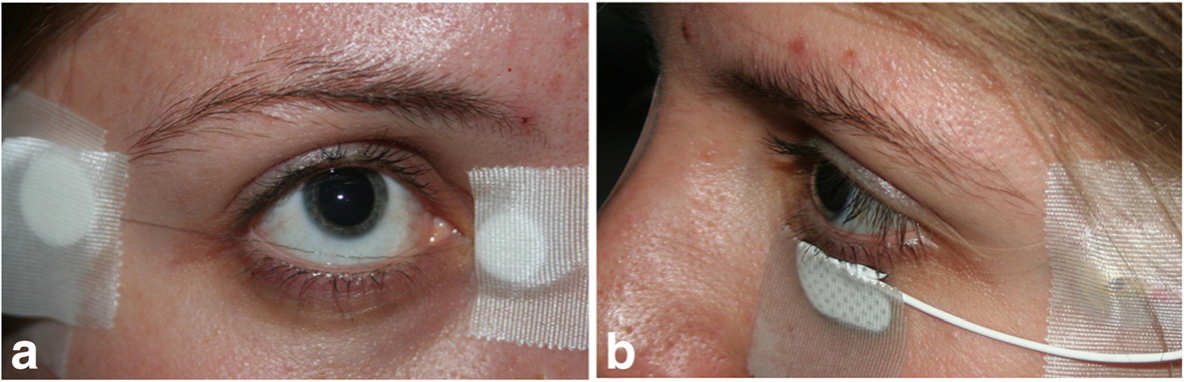
**a** The microfiber (DTL Plus) electrode is placed across the cornea over the lower eyelid (right eye). **b** The Neuroline skin electrode is placed on the surface of the left lower lid (left eye)

The pupils were maximally dilated (7.0–8.0 mm) using Tropicamide 0.5 % and Phenylephrine 1 % eye drops. The cornea was anaesthetized using Proxymetacain Hydrochloride. The skin on the subject‘s forehead was cleaned with an abrasive cream (Everi, Spes medica, Italy) and an electrode cream (Ec2, Astro Med, Inc) was used to adhere the neutral ground electrode to the skin of the forehead. In each subject the single-use silver-impregnated microfiber electrode (DTL Plus Electrode™, Diagnosis LLC, Lowell, MA) was placed across the cornea, above the lower eyelid, draped in the lower fornix, providing thus direct contact with the tear film of the eye. The reference electrode was placed near the lateral cantus and the ground electrode mid frontal. The skin electrode (Neuroline 725, Ambu, Glen Burnie, USA) was placed on the surface of the opposite lower lid and the reference electrode near the ipsilateral outer canthus (Fig. 1a-b). For subjects` comfort the skin electrode was placed on the mid lowed lid shortly before the recording session, after the dark-adaptation was completed.

The hand-held Mini Ganzfeld system (ColorBurst™, Diagnosys LLC, Cambridge, UK) was used for standard full-field ERG stimulus presentation. The Espion system was used for recording and analysis of the data.

The viewing diameter was 3.5” (90 mm) and the view port size was 2.15” (55 mm). A corrective lens was not applied. For each eye, ERGs were recorded once for each stimulus condition. The test order provided by ColorBurst^TM^, Diagnosys LLC software was pre-programmed according to the ISCEV guidelines 2008 [2]. The eyes were tested sequentially, starting with the right eye. The eye on which each electrode type was placed was counterbalanced in regard to the eye tested (1 = OD; 2 = OS) and to the electrode type (DTL = OD; skin electrode = OS). Thus, DTL electrode recordings were performed randomised 16 times OD and 14 times OS versus skin electrode recordings: 13 times OD and 17 times OS. The stimulus flash was activated, once the Mini Ganzfeld stimulator was properly positioned in front of the eye, having had the other eye completely covered. The duration of dark and light adaptation was equal for both eyes.

### Recording session

After 30 min dark-adaptation, rod activity was assessed using series of achromatic flashes against achromatic background according to the ISCEV standards 2008 [2] in the following order: 0.01, 0.03, 0.1, 0.3, 1.0 cd.s/m^2^ (4 milliseconds of duration, each), presented at a rate of 1 flash every 15 s. The standard combined rod-cone response ERG was obtained to 3.0 cd.s/m^2^ achromatic flashes. Following 10 min light adaptation at ambient luminance a light-adapted 3.0 ERG was recorded. The light-adapted 3.0 flicker ERG was obtained to 33-Hz flicker presented at the same stimulus and background luminance as the photopic ERG. In addition to ISCEV standards 2009 [2], four sets of red cone flashes (0.3, 0.5, 0.9, 3.0 cd.s/m^2^) were afterwards superimposed on the constant background and the red cone ERG responses recorded. The retinal responses were bandpass filtered between 0.3 and 300 Hz.

### Data analysis

We analysed amplitudes and implicit times of the dark-adapted ERG (0.01, 0.03, 0.1, 0.3, 1. 0 cd.s/m^2^), the dark-adapted 3.0 ERG, the light-adapted 3.0 ERG, the light-adapted 3.0 flicker response and the red cone ERG (0.3, 0.5, 0.9, 3.0 cd.s/m^2^). The amplitude of the a-wave was measured from the baseline to the a-trough and that of the b-wave: from a-trough to the b-peak by electronic cursors. The light-adapted 3.0 flicker responses were analysed for trough-to-peak amplitude (avoiding the initial response), as well as the corresponding implicit times.

### Statistical analysis

For statistical analysis multivariate ANOVA was applied. Descriptive statistics and frequency tables were obtained to analyse the categorical variables. Results are presented as the mean, standard deviation (±SD) with the corresponding 95 % confidence intervals (95 % CI) and p-values. Full-field ERG parameters: amplitudes and implicit times were treated as dependent variables. The difference between the recordings with DTL electrode versus Neuroline skin electrode was evaluated. Subject tested was taken as a random factor. The eye and the electrode type were taken as fixed factors. Potential interactions between the study groups (electrode and the eye) and the covariates were also included in the regression model. A level of significance of 0.05 was considered relevant.

## Results

Full-field ERG recordings were obtained from a total of 30 control subjects. In 2 subjects the complete ERG recording (Neuroline electrode, OD) was excluded from further statistical analyses due to unreliable data: at the end of the recording period, the ERG responses were not approaching the baseline. That means, in 7 % of cases no measurable ERG could be recorded. From the remaining 28 subjects only the reliable recordings were further analysed: One out of 28 subjects showed a baseline drift of the scotopic 0.01 recording (DTL electrode, OS). Another 7 from the 28 subjects had unsatisfactory scotopic 0.01 recordings on one eye (Neuroline electrode: 2 OD & 4 OS; DTL electrode: 1 OS) and three patients on both eyes (Neuroline electrode: 3 OS; DTL electrode: 3 OD).

**Fig. 2:**
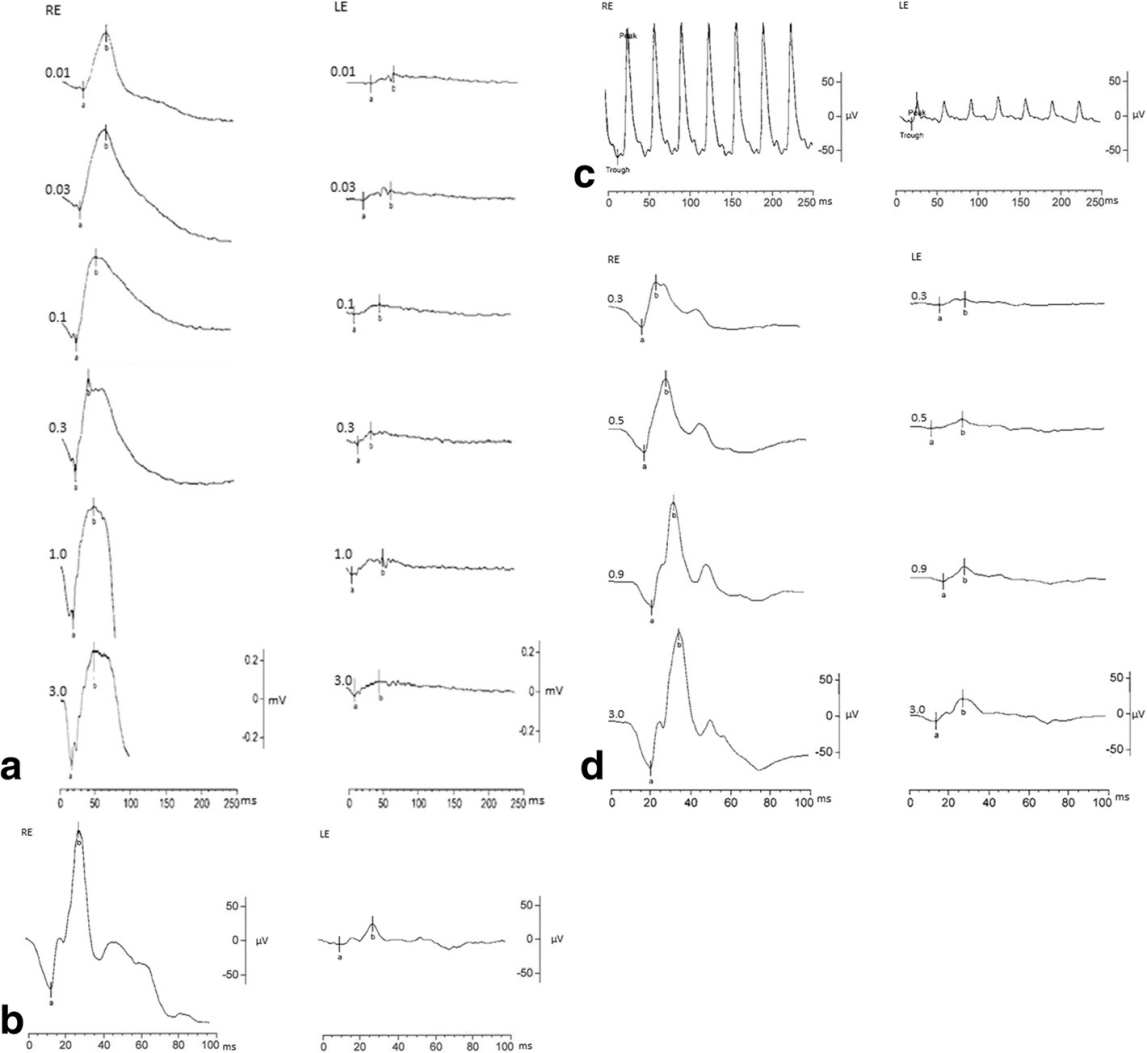
Depicts for comparison representative ERG traces recorded with both electrode types as follows: **a** scotopic, **b** standard flash cone, **c** light-adapted 3.0 flicker, **d** red cone

In general, all evaluated recording values were normally distributed. Responses with Neuroline skin electrode and DTL electrode compared well and showed similar pattern of waveforms, with smaller amplitudes seen with the Neuroline skin electrode (Fig. 2a-d with sample records). Subject’s age did not show statistically significant influence for either of the recordings (*p* > 0.05).

In order to evaluate the effect of flash on retinal adaptation while applying the Mini Ganzfeld ERG hand-held flash stimulator, we took the eye as fixed factor in the multivariate model. Results showed no significant difference between the eyes tested, where the p-values for all stimuli conditions between both eyes were above 0.069.

Intensity series were done for both rod and red cone flash conditions and DTL amplitudes were up to four times larger than skin electrode amplitudes (Fig. 2a, d).

### Analysis of the dark-adapted responses

**Fig. 3:**
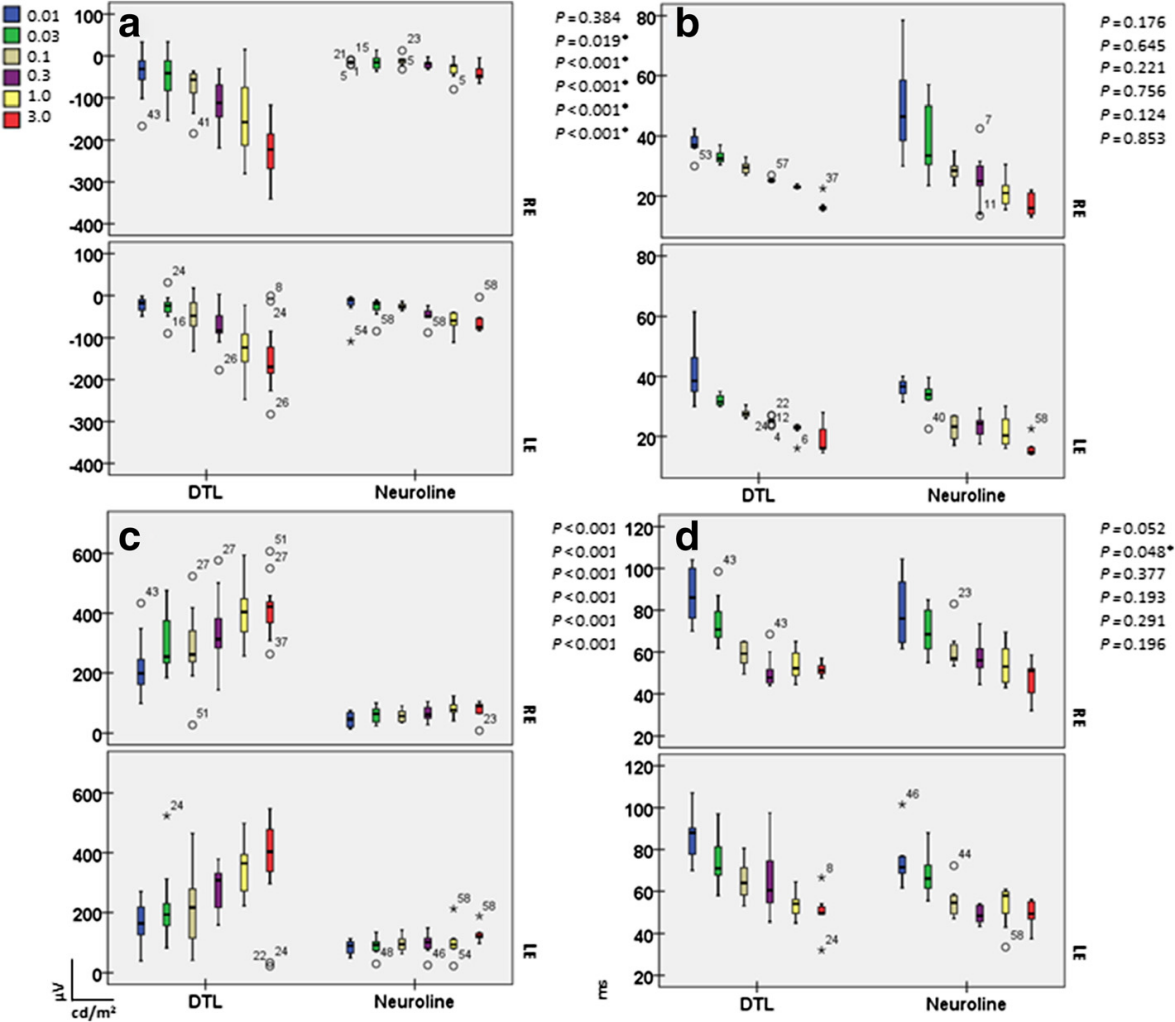
Box plots of the scotopic a-wave amplitudes (**a**), b-wave amplitudes (**c**), a-wave (**b**) and b-wave implicit times (**d**). With increasing luminance the a-wave, as well as the b-wave amplitude got larger resulting in an increase of the DTL: Neuroline amplitude-relationship from 1:1 in lower stimulus intensities to almost 1:4 in higher stimulus intensities. For all box plots, recordings obtained with a DTL electrode are plotted to the left, while recordings obtained with a Neuroline skin electrode are plotted to the right. The box length is the inter-quartile range. The line in bold depicts the median. Values between 1.3 and 3.0 box length represent outliers (open circles) and those with values more than 3.0 box length represent extremes (arrow heads). The respective p-values are shown on the top right hand side of the graphs (multivariate ANOVA)

Examining the scotopic responses, recordings with the Neuroline skin electrode did not show a marked increase in the a- and b-wave amplitudes while increasing intensity (Fig. 2a, right). Figure 3a, c, represent box plots of the scotopic flash a- and b-amplitude responses for both electrode types and for each eye separately. With increasing luminance we recorded the a-wave and the b-wave amplitudes of the DTL electrode up to four times larger, than the corresponding ones of the Neuroline skin electrode (Table 1).

**Table 1:** represents the mean, standard deviation, 95 % confidence interval, as well as their respective p-values (ANOVA based on mixed effects model) of the a- and b-wave amplitudes, as well as the corresponding implicit times of the scotopic ERG for both electrode types

ERG Recordings	Electrodes	Mean ± SD	95 % confidence interval	P-values between eyes	P-values between Elctrodes
A-wave amplitudes					
0.01 cd.s/m^2^	DTL Neuroline	−29.27 ± 37.15 −20.35 ± 23.12	−43.04,−15.50 −35.58,−5.13	0.811	0.384
0.03 cd.s/m^2^	DTL Neuroline	−38.64 ± 41.64 −16.17 ± 21.68	−49.64,−25.01 −28.81,−2.93	0.347	0.019*
0.1 cd.s/m^2^	DTL Neuroline	−60.76 ± 45.91 −17.55 ± 11.54	−71.38,−46.41 −30.57,−2.90	0.289	<0.001*
0.3 cd.s/m^2^	DTL Neuroline	−91.18 ± 50.84 −33.79 ± 18.38	−102.79,−75.34 −46.35,−17.16	0.630	<0.001*
1.0 cd.s/m^2^	DTL Neuroline	−135.63 ± 79.56 −44.21 ± 24.02	−155.86,−111.57 −66.08,−19.00	0.739	<0.001*
3.0 cd.s/m^2^	DTL Neuroline	−197.21 ± 96.78 −48.90 ± 26.47	−219.68,−169.12 −73.05,−20.80	0.120	<0.001*
B-wave amplitudes					
0.01 cd.s/m^2^	DTL Neuroline	197.49 ± 92.05 72.49 ± 42.52	167.65, 227.33 39.51,105.48	0.757	<0.001*
0.03 cd.s/m^2^	DTL Neuroline	254.35 ± 108.02 72.75 ± 34.38	220.85, 278.81 39.70, 100.57	0.306	<0.001*
0.1 cd.s/m^2^	DTL Neuroline	246.02 ± 117.42 80.95 ± 35.54	209.02, 272.11 41.97, 111.88	0.380	<0.001*
0.3 cd.s/m^2^	DTL Neuroline	308.60 ± 105.02 90.03 ± 45.73	274.70, 330.52 56.32, 115.67	0.260	<0.001*
1.0 cd.s/m^2^	DTL Neuroline	371.47 ± 92.31 90.16 ± 39.05	341.58, 392.78 61.35, 115.78	0.213	<0.001*
3.0 cd.s/m^2^	DTL Neuroline	394.34 ± 133.71 97.41 ± 40.98	354.93, 428.03 55.84, 131.40	0.460	<0.001*
A-wave implicit times					
0.01 cd.s/m^2^	DTL Neuroline	38.98 ± 7.75 43.28 ± 12.95	34.73, 43.22 38.59, 47.97	0.335	0.176
0.03 cd.s/m^2^	DTL Neuroline	34.17 ± 9.15 34.92 ± 8.66	31.07, 37.62 31.99, 38.88	0.640	0.645
0.1 cd.s/m^2^	DTL Neuroline	28.14 ± 2.09 26.50 ± 4.91	26.80, 29.35 25.50, 28.31	0.011*	0.221
0.3 cd.s/m^2^	DTL Neuroline	25.30 ± 0.87 24.82 ± 5.47	23.87, 26.71 23.46, 26.48	0.407	0.756
1.0 cd.s/m^2^	DTL Neuroline	22.97 ± 2.13 21.44 ± 4.68	21.63, 24.31 20.02, 22.87	0.986	0.124
3.0 cd.s/m^2^	DTL Neuroline	17.81 ± 3.59 18.07 ± 4.61	16.31, 19.45 16.47, 19.72	0.416	0.853
B-wave implicit times					
0.01 cd.s/m^2^	DTL Neuroline	86.43 ± 12.00 77.46 ± 15.61	80.37, 92.50 70.75, 84.17	0.576	0.052
0.03 cd.s/m^2^	DTL Neuroline	73.17 ± 12.93 66.12 ± 12.45	68.61, 78.10 61.44, 71.41	0.985	0.048*
0.1 cd.s/m^2^	DTL Neuroline	61.48 ± 10.18 58.80 ± 12.43	57.69, 66.00 54.48, 63.69	0.671	0.377
0.3 cd.s/m^2^	DTL Neuroline	56.69 ± 14.24 52.88 ± 10.47	51.71, 58.42 48.90, 56.03	0.237	0.193
1.0 cd.s/m^2^	DTL Neuroline	55.08 ± 8.43 52.46 ± 9.42	51.82, 58.34 49.02, 55.89	0.954	0.291
3.0 cd.s/m^2^	DTL Neuroline	51.64 ± 5.62 47.99 ± 11.18	48.28, 54.94 45.05, 51.93	0.197	0.196

Statistically significant *p*-values (<0.05) are labeled with asteriks

That is, the Neuroline skin electrode responses remained so small, that changes could barely be detected. For instance, the mean amplitude of the standard combined (3.0 cd.s/m^2^) a-wave response was -48.90 μV (SD: ±26.47) with the Neuroline skin electrode versus -197.20 μV (SD: ±96.78) with the DTL electrode, (*p* < 0.001). For the b-wave, the respective values of the scotopic 3.0 were 97.41 μV (SD: ±40.98), versus 394.33 μV (SD: ±133.71) (*p* < 0.001). Only for stimulus intensity of 0.01 cd.s/m^2^ there was no statistically significant difference whether the a-wave responses were obtained with the skin electrode or with the DTL electrode (*p* = 0.384, multivariate ANOVA).

Implicit times of the dark-adapted ERGs showed no statistically significant difference for the a-wave in all stimulus intensities between both electrode types (Fig. 3c). For the b-wave, the difference between both electrodes reached the significance level only at intensity 0.03 cd.s/m^2^ (*p* = 0.048) (Fig. 3d).

### Analysis of the light adapted responses

**Fig. 4:**
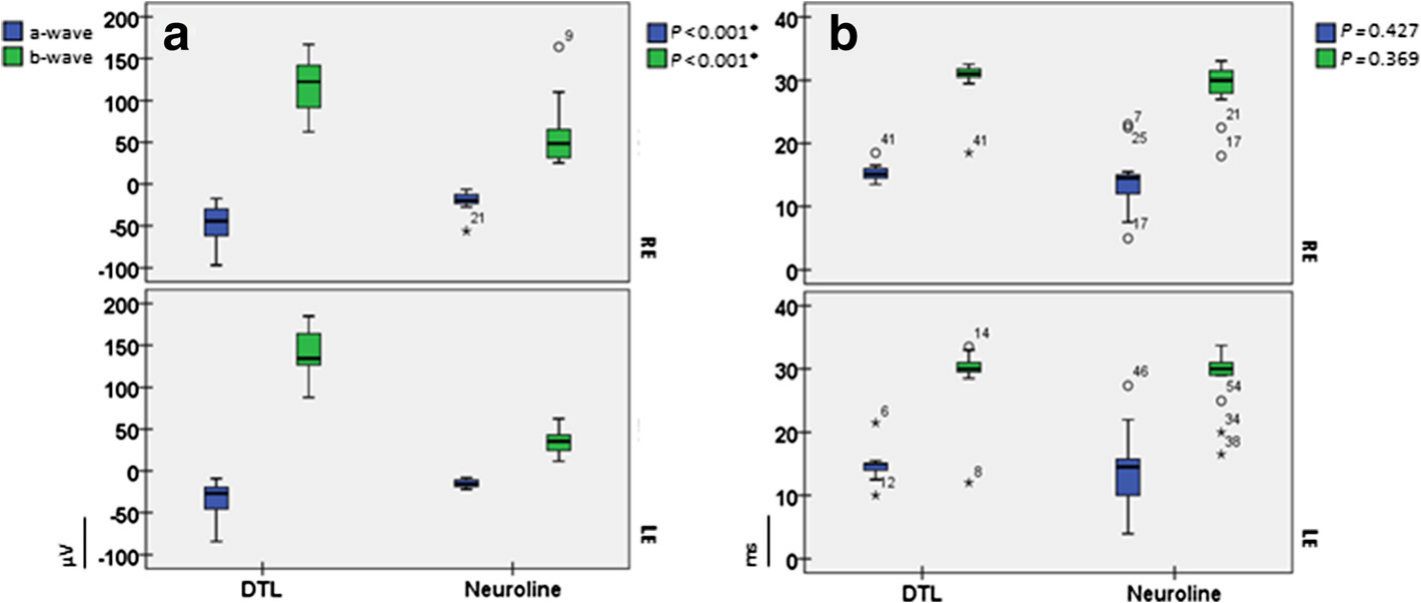
Box plots of the light-adapted 3.0 responses. **a** depicts box plots of the a-, and b-wave amplitudes, whereas **b** depict box plots of the a-, and b-wave implicit times recorded with both electrode types. For all box plots, recordings obtained with a DTL electrode are plotted to the left, while recordings obtained with a Neuroline skin electrode are plotted to the right. The box length is the inter-quartile range. The line in bold depicts the median. Values between 1.3 and 3.0 box length represent outliers (open circles) and those with values more than 3.0 box length represent extremes (arrow heads). The respective p-values are shown on the top right hand side of the graphs (multivariate ANOVA)

**Fig. 5:**
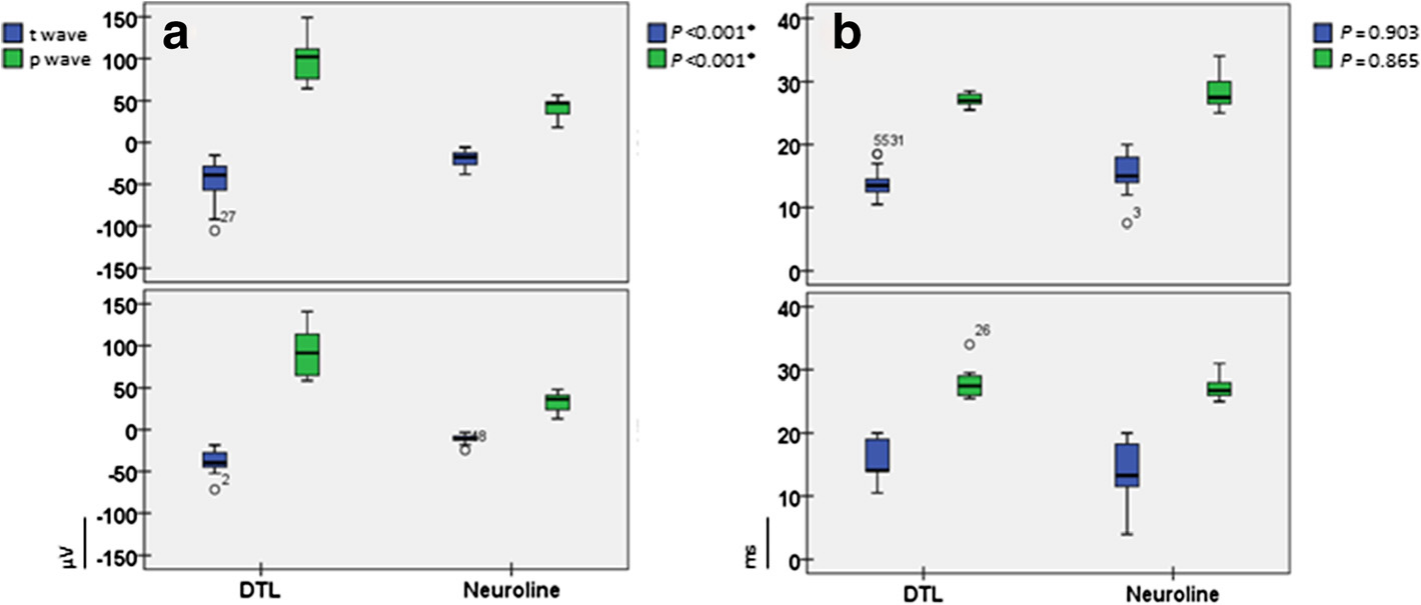
Shows the light-adapted 3.0 flicker box plots. The trough- and peak-amplitudes are presented in **a**. The data of the corresponding implicit times are shown in **b.** For all box plots, recordings obtained with a DTL electrode are plotted to the left, while recordings obtained with a Neuroline skin electrode are plotted to the right. The box length is the inter-quartile range. The line in bold depicts the median. Values between 1.3 and 3.0 box length represent outliers (open circles) and those with values more than 3.0 box length represent extremes (arrow heads). The respective p-values are shown on the top right hand side of the graphs (multivariate ANOVA)

**Fig. 6:**
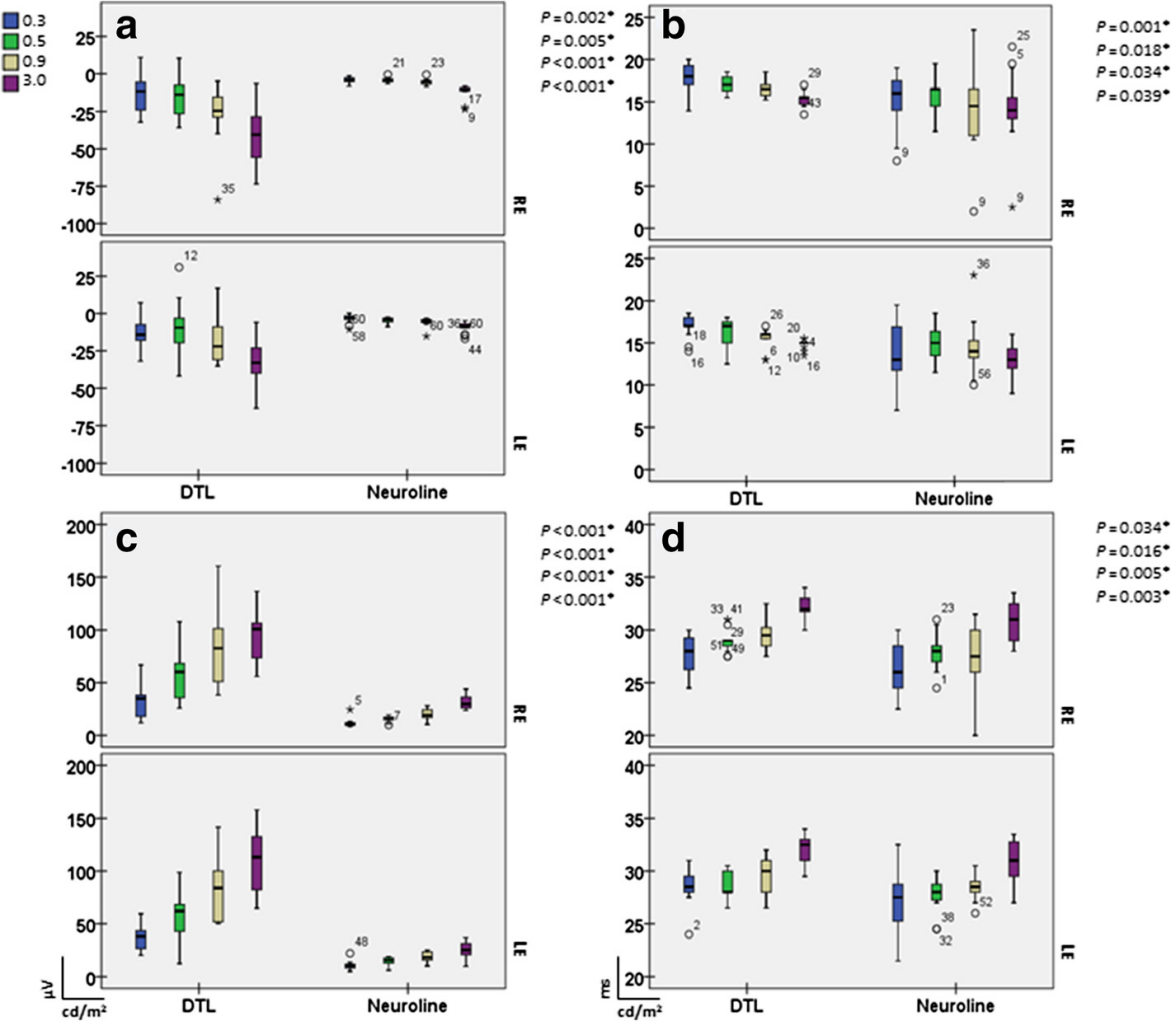
Respective box plots for a-wave amplitudes (**a**), b-wave amplitudes (**c**), a-wave (**b**) and b-wave implicit times (**d**) of the red cone response. In analogy to the scotopic response, with increasing stimulus intensity the a- as well as the b-red cone response got larger in the DTL electrode recordings compared to the Neuroline skin electrode recordings. A novel finding is the implicit time delay of the red a- and b-waves with the DTL electrode when compared to the recordings with the Neuroline skin electrode (Table 4). For all box plots, recordings obtained with a DTL electrode are plotted to the left, while recordings obtained with a Neuroline skin electrode are plotted to the right. The box length is the inter-quartile range. The line in bold depicts the median. Values between 1.3 and 3.0 box length represent outliers (open circles) and those with values more than 3.0 box length represent extremes (arrow heads). The respective p-values are shown on the top right hand side of the graphs (multivariate ANOVA)

As expected, amplitudes of a-waves, as well as of b-waves of the standard flash cone and red cone responses were significantly smaller when recorded with the Neuroline skin electrode (*p* ≤ 0.005, Figs. 4a, 6a, c). The same held true for the trough- and peak- amplitudes of the 30Hz flicker response (*p* < 0.001, Fig. 5a).

### Analyses of the standard flash cone response (light-adapted 3.0)

While the light-adapted 3.0 amplitudes differed between both electrode types for a-wave, as well as for the b-wave amplitudes (*p* < 0.01), this did not hold true for the a- and b-wave amplitude implicit times (*p* ≥ 0.369, Fig. 4a, b). Here, the mean a-wave and b-wave amplitudes recorded with the DTL electrode were -42.85 (SD: ±24.33) and 124.95 (SD: ±32.90), whereas with the Neuroline, they were -17.76 (SD: ±9.36) and 46.60 (SD: ±31.12), respectively (Table 2).

**Table 2:** shows the mean, standard deviation, 95 % confidence interval, as well as their respective p-values (ANOVA based on mixed effects model) of the a- and b-wave amplitudes, as well as the corresponding implicit times of the photopic flash ERG for both electrode types

ERG Recordings	Electrodes	Mean ± SD	95 % confidence interval	P-values between eyes	P-values between elctrodes
A-wave amplitudes					
	DTL Neuroline	−42.85 ± 24.33 −17.76 ± 9.36	−49.06,−35.39 −24.91,−11.04	0.069	<0.001*
B-wave amplitudes					
	DTL Neuroline	124.95 ± 32.90 46.60 ± 31.12	114.68, 137.45 35.91, 59.02	0.868	<0.001*
A-wave implicit times					
	DTL Neuroline	15.03 ± 1.89 14.12 ± 5.39	13.47, 16.53 12.58, 15.68	0.735	0.427
B-wave implicit times					
	DTL Neuroline	29.76 ± 4.28 28.63 ± 4.41	28.05, 31.35 26.96, 30.31	0.588	0.369

Statistically significant *p*-values (<0.05) are labeled with asteriks

### Analysis of 30 Hz ERG (light-adapted 3.0 flicker)

As exemplified in Fig. 5b, the implicit times of the 30 Hz flicker response did not differ significantly between the electrode types (*p* = 0.865). For instance, the mean P-implicit time for the DTL electrode was 27.58 ms (SD: ±1.64), whereas for the Neuroline: 27.50 ms (SD: ±2.15) (Table 3).

**Table 3:** presents the mean, standard deviation, 95 % confidence interval, as well as their respective p-values (ANOVA based on mixed effects model) of the peak- and trough- amplitudes, as well as the corresponding implicit times of the 30Hz ERG for both electrode types

ERG Recordings	Electrodes	Mean ± SD	95 % confidence interval	P-values between eyes	P-values between elctrodes
T-wave amplitudes					
	DTL Neuroline	−42.54 ± 20.92 −14.85 ± 8.24	−47.99,−36.20 −21.20,−9.06	0.091	<0.001*
P-wave amplitudes					
	DTL Neuroline	97.64 ± 26.46 37.15 ± 11.69	89.54, 104.85 29.56, 45.31	0.184	<0.001*
T-wave implicit times					
	DTL Neuroline	14.75 ± 2.80 14.96 ± 3.39	13.73, 16.01 13.80, 16.14	0.333	0.903
P-wave implicit times					
	DTL Neuroline	27.58 ± 1.64 27.50 ± 2.15	26.93, 28.32 26.82, 28.25	0.633	0.865

Statistically significant *p*-values (<0.05) are labeled with asteriks

### Analysis of the red cone responses

In addition to ISCEV standards 2008 [2] for full-field ERG recordings, red cone ERG responses were recorded. As in the scotopic response, a- and b-wave amplitudes of the red cone response did not change much with different stimulus intensity when recorded with the Neuroline skin electrode, whereas with the DTL electrode the amplitudes got higher with increasing stimulus intensity (*p* ≤ 0.005; Fig. 6a, c). This led to an almost 1:3 DTL amplitude-relationship in higher stimulus intensities when compared to a 1:1 ratio at lower stimulus intensities (Table 4).

**Table 4:** shows the mean, standard deviation, 95 % confidence interval, as well as their respective p-values (ANOVA based on mixed effects model) of the a- and b-wave amplitudes, as well as the corresponding implicit times of the photopic red ERGs for both electrode types

ERG Recordings	Electrodes	Mean ± SD	95 % confidence interval	P-values between eyes	P-values between elctrodes
A-wave amplitudes					
0.3 cd.s/m^2^	DTL Neuroline	−11.46 ± 12.56 -3.89 ± 2.72	−14.96,−8.15 −7.31,−0.50	0.787	0.002*
0.5 cd.s/m^2^	DTL Neuroline	−14.44 ± 17.73 −4.49 ± 1.84	−18.56,−9.37 −8.99, 0.08	0.185	0.005*
0.9 cd.s/m^2^	DTL Neuroline	−20.89 ± 18.35 −5.48 ± 2.46	−25.16,−15.56 −10.28,−0.68	0.250	<0.001*
3.0 cd.s/m^2^	DTL Neuroline	−36.30 ± 17.36 −10.43 ± 4.41	−40.53,−31.14 −15.29,−5.77	0.082	<0.001*
B-wave amplitudes					
0.3 cd.s/m^2^	DTL Neuroline	35.41 ± 15.40 10.99 ± 3.92	31.41, 39.80 6.88, 15.28	0.784	<0.001*
0.5 cd.s/m^2^	DTL Neuroline	57.06 ± 23.26 14.94 ± 3.36	50.64, 63.17 8.81, 21.17	0.668	<0.001*
0.9 cd.s/m^2^	DTL Neuroline	83.93 ± 31.70 19.46 ± 4.83	75.31, 92.33 11.03, 28.05	0.818	<0.001*
3.0 cd.s/m^2^	DTL Neuroline	100.75 ± 29.05 28.16 ± 7.47	94.02, 109.42 20.55, 36.18	0.241	<0.001*
A-wave implicit times					
0.3 cd.s/m^2^	DTL Neuroline	17.22 ± 1.79 14.46 ± 3.51	16.17, 18.23 13.52, 15.58	0.231	0.001*
0.5 cd.s/m^2^	DTL Neuroline	16.62 ± 1.51 15.34 ± 2.19	15.86, 17.28 14.67, 16.06	0.210	0.018*
0.9 cd.s/m^2^	DTL Neuroline	15.97 ± 1.21 14.27 ± 3.98	14.82, 17.02 13.14, 15.34	0.816	0.034*
3.0 cd.s/m^2^	DTL Neuroline	15.02 ± 0.84 13.56 ± 3.45	14.07, 15.94 12.66, 14.55	0.241	0.039*
B-wave implicit times					
0.3 cd.s/m^2^	DTL Neuroline	27.92 ± 1.85 26.72 ± 2.66	27.14, 28.84 25.84, 27.54	0.245	0.034*
0.5 cd.s/m^2^	DTL Neuroline	28.76 ± 1.21 27.80 ± 1.61	28.21, 29.29 27.28, 28.34	0.657	0.016*
0.9 cd.s/m^2^	DTL Neuroline	29.50 ± 1.54 28.00 ± 2.41	28.76, 30.26 27.20, 28.70	0.410	0.005*
3.0 cd.s/m^2^	DTL Neuroline	32.22 ± 1.27 30.87 ± 1.93	31.62, 32.83 30.25, 31.48	0.828	0.003*

Statistically significant *p*-values (<0.05) are labeled with asteriks

For instance, the mean amplitude of the red cone 3.0 cd.s/m^2^ a-wave response was -10.43 μV (SD: ±4.41) with the Neuroline skin electrode versus -36.30 μV (SD: ±17.36) with the DTL electrode, (*p* < 0.001). For the b-wave, the respective values of the red cone 3.0 ERG were 28.16 μV (SD: ±7.47), versus 100.75 μV (SD: ±29.05) (*p* < 0.001).

Figure 6b and d represent box plots of the red cone a- and b-wave implicit times for both electrode types. Noticeably, in all stimuli intensities, the a- and b-wave implicit time of the red cone responses differed in a statistically significant way between electrodes (*p* ≤ 0.039). The light adapted implicit times of the red a- and b-waves were slightly faster for the skin electrode recordings. Here for instance, the mean implicit time of the red cone response (3.0 cd.s/m^2^) for the a-wave was: 13.56 ms (SD: ±3.45) for the Neuroline skin electrode and 15.02 ms (SD: ±0.84) for the DTL electrode, (*p* = 0.039). For the b-wave (3.0 cd.s/m^2^) the respective values were 30.87 ms (SD: ±1.93) and 32.22 ms (SD: ±1.27), (*p* = 0.003).

### Mean values Standard deviation of DTL microfiber electrode versus Neuroline skin electrode

Interestingly, for all stimuli intensities, the a- and the b-wave amplitude recordings with the DTL electrode showed higher standard deviation (SD), when compared to the Neuroline skin electrode (Tables 1, 2, 3 and 4). For instance, the SD of a-wave amplitudes of scotopic ERG varied for the DTL electrode recordings between 37.15 ms and 96.78 ms, and of the b-wave amplitudes between 92.05 ms and 133.71 ms, whereas for the corresponding recordings with the Neuroloine skin electrode SD varied between 11.54 ms and 26.47 ms, respectively between 34.38 ms and 45.73 ms (Table 1). The same held true for the amplitudes of the light adapted responses (Tables 2, 3 and 4). The SD of the respective implicit times did not differ significantly between the electrode types.

## Discussion

Electrophysiological testing for children and infants still remains challenging, in particular when the recording is accompanied by sedation or anesthesia. Different approached has been discussed between ISCEV members, as: running the standard ISCEV ERG protocol only on one eye in bilateral pathologies; performing photopic ERGs on one eye while dark adaptating the second eye; checking the cones immediately and watching the recovery of rods then; or using melatonin for sleep-induction for ERG instead of sedation or anesthesia.

Given the increased compliance of infants with the positioning of skin electrodes, we aimed to compare responses obtained using the DTL microfiber and the Neuroline surface electrode types in the Mini Ganzfeld ERG, applying the stimulation of modified ISCEV standard protocol (2008) [2] on healthy adult subjects. To the best of our knowledge, this study is the first to report the comparison of ERG waveforms between two non-cornea contact electrodes, the DTL microfiber and the Neuroline surface electrode in the Mini Ganzfeld hand-held ERG device. Therefore, the data cannot be compared with previous studies.

In this study the data were collected on adults, aged between 19 and 61 years. Although age may influence the ERG recording and should be taken into account [6, 24, 25], within our examined age group the results showed no statistically significant influence of age (*p* > 0.005).

Overall, responses with the Neuroline skin electrode and the microfiber DTL electrode showed the same waveform characteristics and compared well. This is not an unexpected finding, as previous studies using different electrode types (skin, contact-corneal, Burian-Allen, Dawson-Trick-Litzkow (DTL), PVA-gel electrodes) showed also similar waveforms [6, 11, 13, 15, 16, 26–29].

Contact lens electrodes are reported to produce higher amplitudes than DTL electrodes [27, 28]. Skin electrodes produced almost three times smaller recordings when compared to HK-loop electrodes [30]. Also, skin electrode recordings were shown to be almost 4.5 times smaller than those recorded with Gold Foil or with Burian-Allen electrodes [11, 16]. Studies on pattern ERG and PhNR have been shown up to 60 % reduction of the responses when the skin electrode recordings were compared to the DTLs [29, 31, 32]. Therefore, it is expected that the Neuroline recordings on the Mini Ganzfeld should be smaller than the DTL recordings.

In our study, contact DTL microfiber electrodes produced in overall larger ERGs than the skin surface electrodes. This amplitude difference was more pronounced for the b-wave amplitudes. Under scotopic conditions amplitudes of the skin electrode recordings are reported to be almost one fourth compared to the DTLs [31]. An interesting finding in the present study, for the dark-adapted but also for the red-ERGs, was that a-wave as well as b-wave responses with the Neuroline skin electrode were of almost constant size for all stimulus energies, whereas responses with the DTL electrode increased steadily once the light intensity increased (Figs. 2a, d, 3a, c, 6a, c). This led to an almost 1:4 skin:DTL amplitude-relationship in higher stimulus intensities for the dark-adapted ERG and to 1:3 for the red-ERG compared to a 1:1 ratio at lower stimulus intensities.

A difference in the amplitude of the DTL versus the JET electrode for the red-stimuli and more pronounced for the dark-adapted stimuli (3.0 cd.s/m^2^) has already been reported, although the results did not reach the significance level [28]. We also found in our study significant differences not only in the amplitude, but also in the implicit time, when the DTL electrode recording was compared against the Neuroline one. Also, dark-adapted and red cone ERG implicit times for both electrodes got shorter with luminance increase. Hence, for the dark-adapted ERG, only the dark-adapted 0.03 cd.s/m^2^ intensity implicit time showed a statistically significant difference between both electrode types (*p* = 0.048). This is due to the prolonged b-wave implicit times of the DTL electrode recordings*.* For all intensities, red cone ERG recordings seemed to be significantly faster with the Neuroline skin electrode (*p* ≤ 0.039). Even with no available literature data on the shortened implicit time for the skin electrode of the red ERGs, this finding has been mentioned by other ISCEV members (in personal communications), as possibly related to the closer positioning of the active skin electrode to the posterior pole of the eye.

### Higher standard deviation of amplitudes in the DTL recording

When compared to the skin electrode, we observed a relatively higher standard deviation of the DTL recording amplitudes. The result is consistent with some previous studies, which reported also a great variability in amplitudes (27 %) and in implicit times using DTL electrodes (almost 31 %) [28]. The study of Mortlock et al. [29] also showed higher standard deviation for the amplitudes of the photopic negative response recorded with the DTL electrode, when compared to the skin electrode. Based on this finding, one can speculate the ERG recordings with DTL to be much more sensitive to blinking artefacts evoked by high intensity flashes, thus producing higher standard deviations of amplitudes and latencies.

Also, as it has been previously reported, the position of the DTL electrode in the lower fornix/conjunctival sac varies from patient to patient [11, 28], which as a consequence leads to increased session-to-session and inter-individual variability. In addition, the DTL recording might be influenced by orbicular muscle artefacts produced by the canthal positioning of the reference electrode. Recordings with the Neuroline electrodes are further away from the cornea, explaining smaller amplitudes and thus, a reduced signal-to-noise ratio, especially at low intensities where the signal is lower. Although more stably positioned on the eyelid, the recordings obtained with these skin electrodes are also dependent on whether the eye is directed straight ahead or in other gaze positions such as upwards and thus away from the electrode. DTL electrodes show here their priority for application in the ERG lab, being better tolerated from animal models. In humans, some useful approaches for optimal skin electrode recording have been discussed, as performing recording in lateral gaze position or placing the electrode on the temporal part of the lower lid, close to the lateral canthus [14].

In conclusion, producing overall larger ERGs, the DTL microfiber electrode recording is superior to the Neuroline skin electrode recording, when an accurate diagnosis of retinal dysfunction is indicated. Here, the proper positioning of the DTL electrode, the orbicular muscle artefacts and the blinking artefacts should be taken into account, when a precise follow up is necessary. However, skin electrode use frequently results in better compliance with infant ERG recordings. Therefore, it is quite significant to confirm that both electrode types allow successful full-field ERG recordings in humans.

Nevertheless, we ought to point out some limitations of our study: we have not recorded 10.0 cd.s/m^2^ rod-cone ERG responses, included nowadays in the updated ISCEV 2015 standard [3], as the acquisition of the data was done between January 2013 and December 2013 [2]. Further studies would be necessary, in order to test the recording characteristics of the 10.0 cd.s/m^2^ rod-cone ERG responses.

One other limitation of the study was a failure to record a response in 2/30 cases, which might be an important issue, especially when evaluating a pediatric patient or monitoring for retinal toxicity, as for instance for Sabril toxicity. In addition, evaluation of inter-visit variability of recordings with DTL- and skin electrodes were not part of the applied protocol.

In regard to the effect of flash stimulation on retinal adaptation while applying the Mini Ganzfeld ERG hand-held flash stimulator, we found no significant difference between the eyes tested for any stimuli conditions. That is, the closer positioning of the hand-held stimulator to the eye seems to be able to keep the retinal illumination of both eyes constant.

Last but not least, separate reference data for adults and children for both electrode types and the Mini Ganzfeld ERG hand-held flash stimulator remain mandatory.

## Conclusions

Comparison of full-field ERG recordings with microfiber DTL and Neuroline skin electrodes showed that DTL electrodes produce larger ERGs. Hence, we provide evidence that both electrode types allow successful full-field ERG recording, although separate normative data for both electrodes are necessary.

## Abbreviations

dark-adapted 3.0 ERG, formerly “maximal or standard combined rod-cone response”; dark-adapted ERG, formerly “rod response”; DTL electrode, Dawson-Trick-Litzkow electrode; ERG, electroretinogram; full-field ERG, full-field electroretinogram; ISCEV, International Society for Clinical Electrophysiology of Vision; light-adapted 3.0 ERG, formerly “single-flash cone response”; light-adapted 3.0 flicker ERG, formerly “30 Hz flicker”; OD, right eye; OS, left eye; red cone ERG, red cone electroretinogram.
